# Supplementary material for: Upper airway gene expression shows a more robust adaptive immune response to SARS-CoV-2 in children
Source: Nat Commun. 2022 Jul 8;13:3937. doi: 10.1038/s41467-022-31600-0 (PMC9263813; doi:10.1038/s41467-022-31600-0)
Supplement: Supplementary file 1 — Supplementary Information [file 41467_2022_31600_MOESM1_ESM.pdf]

Supplementary Figures

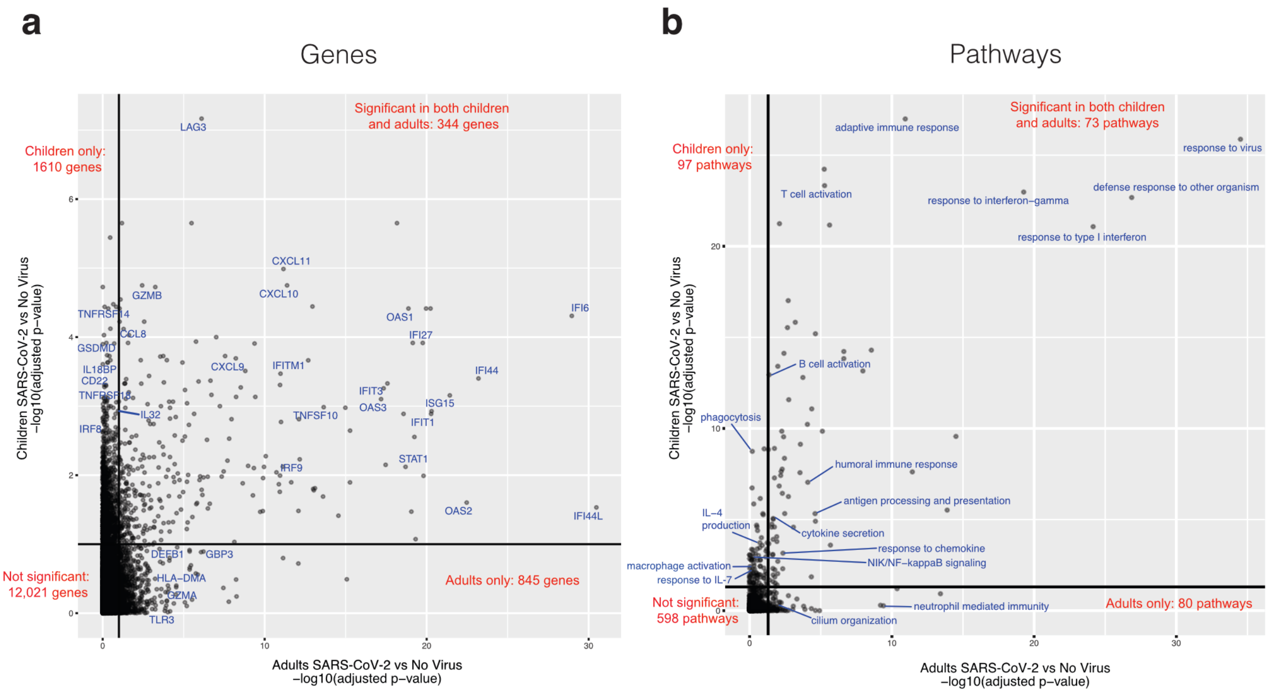

Supplementary Figure 1: Overlap of significant genes and pathways in the differential expression analyses between the SARS-CoV-2 and No Virus groups in adults and children. a) Scatter plot of -$\log_{10}$(adjusted p-value) for individual genes from the differential expression analyses of the SARS-CoV-2 and No Virus groups in the adult (x-axis) and pediatric (y-axis) cohorts. P-values derive from a moderated *t*-statistic and Benjamini-Hochberg adjusted. Shown are all genes included in both analyses (n=14,820). Black lines indicate the threshold of adjusted p-value < 0.1. The number of genes in each quadrant is indicated and selected immune-related genes are labeled. Adults-No Virus n=81, Adults-SARS-CoV-2 n=45, Children-No Virus n=34, Children-SARS-CoV-2 n=38. **b)** Scatter plot of -$\log_{10}$(adjusted p-value) for pathways from the gene set enrichment analyses based on the SARS-CoV-2 versus No Virus comparisons in the adult (x-axis) and pediatric (y-axis) cohorts. P-values are based on an adaptive, multilevel splitting Monte Carlo approach and Benjamini-Hochberg adjusted. Shown are all pathways included in both analyses (n=848 pathways). Black lines indicate the threshold of adjusted p-value < 0.05. The number of pathways in each quadrant is indicated and selected immune-related pathways are labeled.

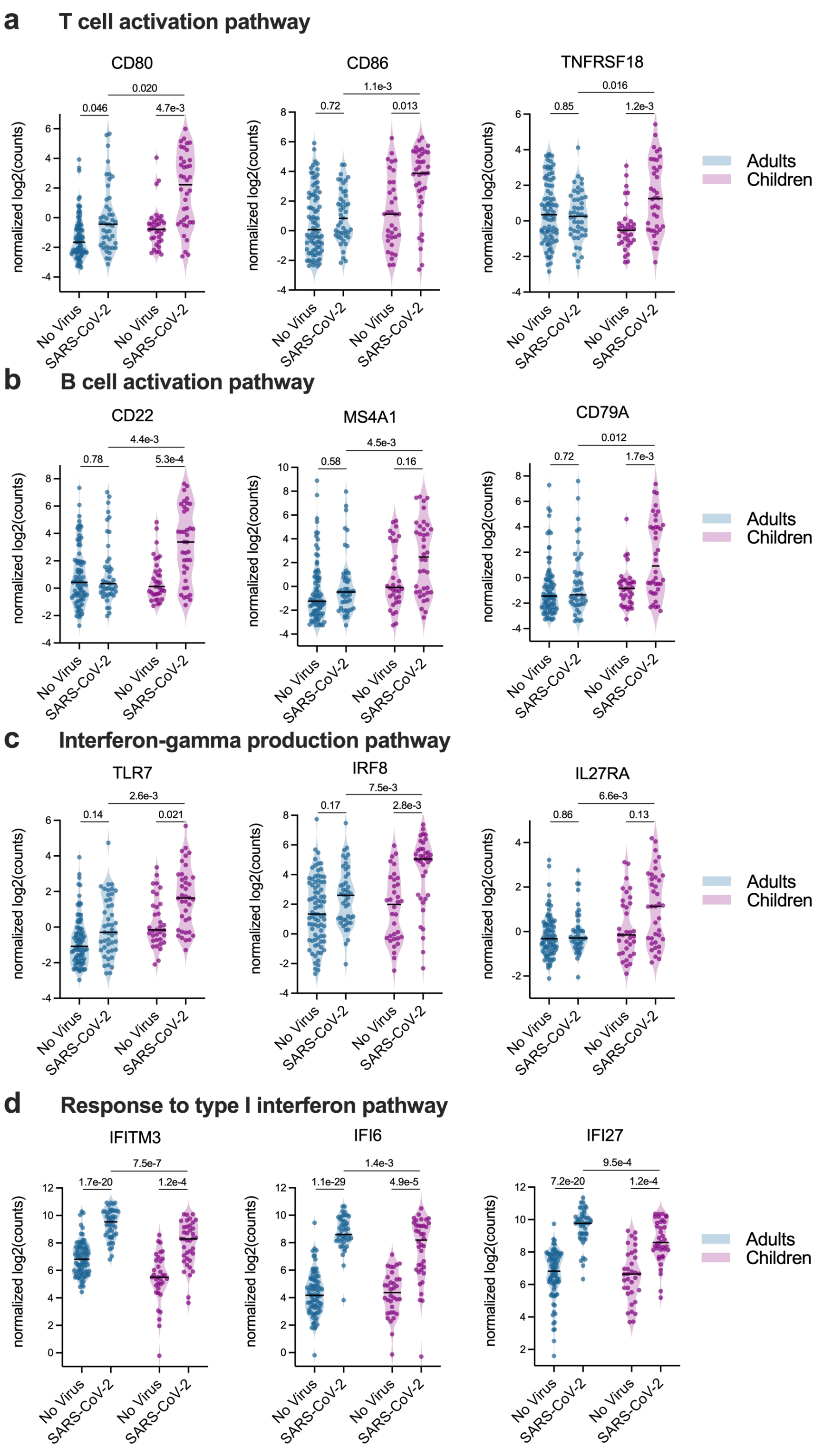

Supplementary Figure 2: Boxplots of selected leading-edge genes for pathways of interest. Boxplots of normalized gene expression for selected leading-edge genes with large fold-changes in the comparison of adults and children with SARS-CoV-2 infection. Color indicates the age

cohort. Black lines denote the median. P-values from the respective differential expression analysis, based on a moderated *t*-statistic and Benjamini-Hochberg adjusted, are indicated. **a)** B cell activation pathway. **b)** T cell activation pathway. **c)** IFNγ production pathway. **d)** Response to type I interferon pathway. Adults-No Virus n=81, Adults-SARS-CoV-2 n=45, Adults-Other Virus n=28, Children-No Virus n=34, Children-SARS-CoV-2 n=38, Children-Other Virus n=11.

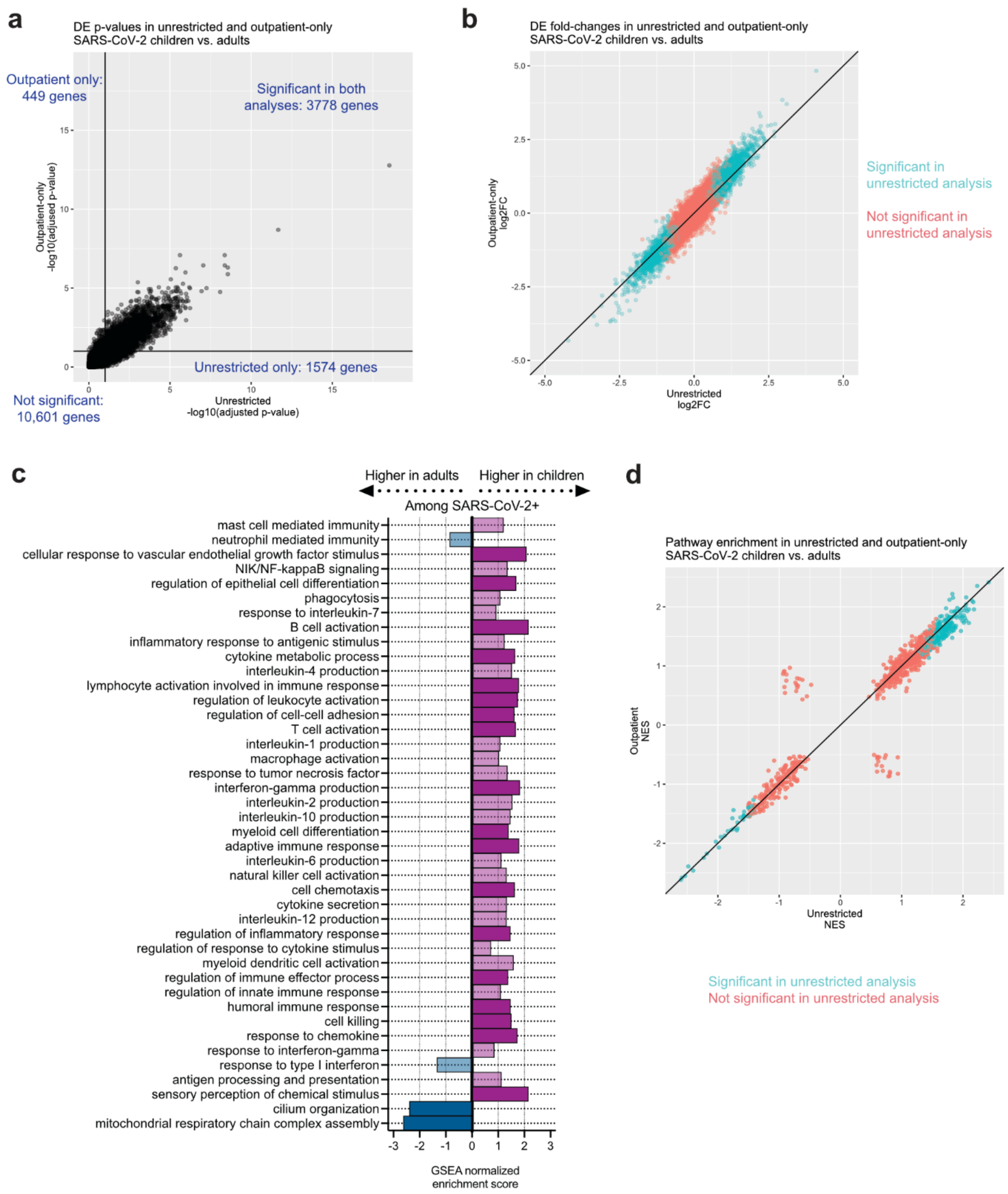

Supplementary Figure 3: Overlap between the unrestricted and outpatient-only versions of the differential expression and gene set enrichment analyses comparing children and adults with SARS-CoV-2 infection.

a) Scatter plot of -$\log_{10}$(adjusted p-value) for individual genes in the unrestricted analysis (x-axis) and outpatient-only analysis (y-axis). P-values are based on a moderated *t*-statistic and

Benjamini-Hochberg adjusted. Black lines indicate the threshold of adjusted p-value < 0.1. The number of genes in each quadrant is indicated. Unrestricted Adults-SARS-CoV-2 n=45, Unrestricted Children-SARS-CoV-2 n=38, Outpatient Adults-SARS-CoV-2 n=24, Outpatient Children-SARS-CoV-2 n=30. **b)** Scatter plot of log_2(fold-change). Genes are colored by whether they were significant in the unrestricted analysis (adjusted p-value < 0.1). **c)** Normalized enrichment scores for the same GO terms shown in **Figure 2** while using the results from the outpatient-only analysis. Color indicates the age cohort. Dark color bars represent pathways that reached statistical significance (adjusted p-value < 0.05). **d)** Scatter plot of pathway normalized enrichment scores (NES) in the unrestricted (x-axis) and outpatient-only (y-axis) gene set enrichment analysis. Pathways are colored by whether they were significant in the unrestricted analysis (adjusted p-value < 0.05).

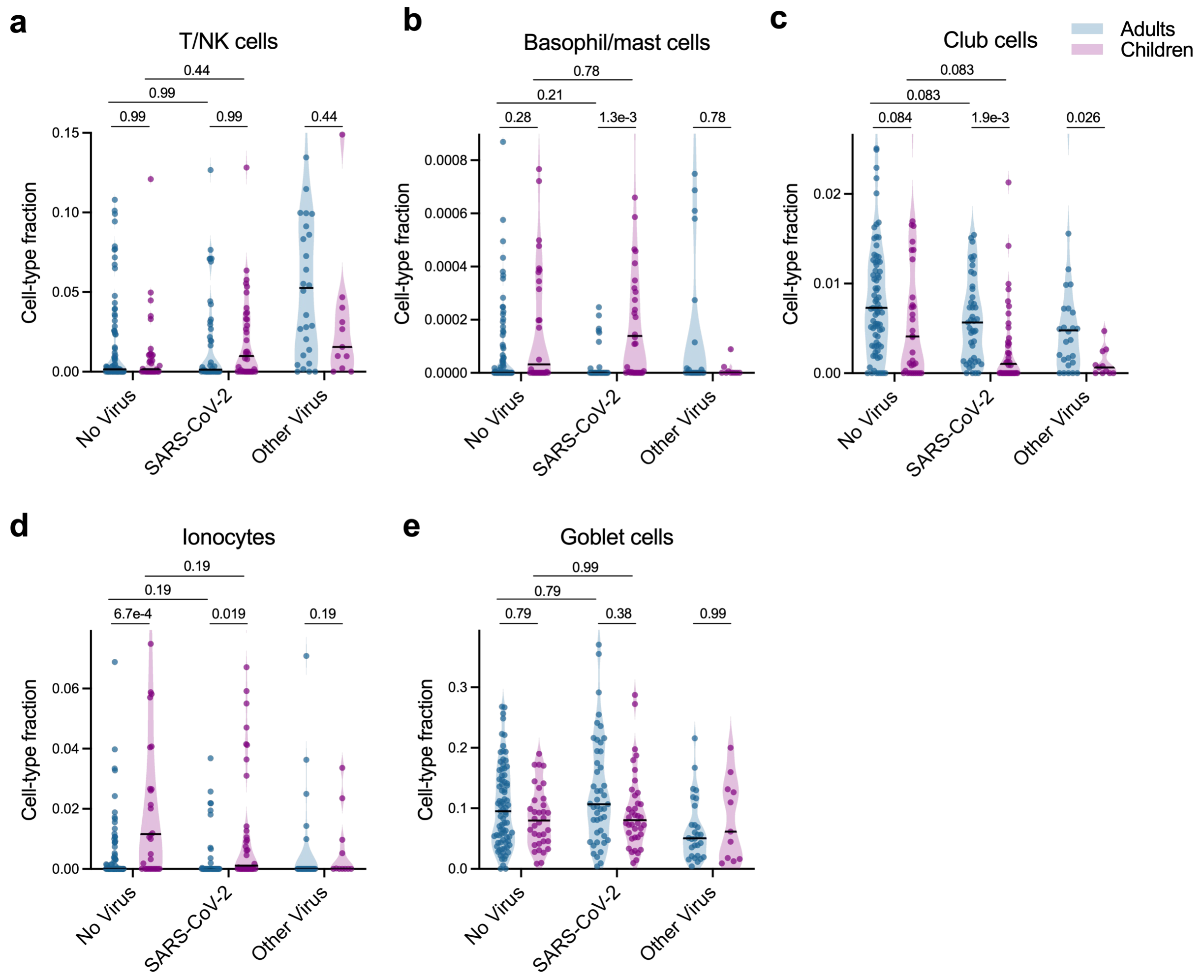

Supplementary Figure 4: Additional cell types from the cell-type proportions analysis.

a-e) Color indicates the age cohort. Black lines denote the median. The y-axis in each panel was trimmed at the maximum value among all groups of 1.5*IQR above the third quartile, where IQR is the interquartile range. For each cell type, we formally compared each viral status group between the two age cohorts as well as the No Virus and SARS-CoV-2 groups within each age cohort. Pairwise comparisons were performed with a two-sided Mann-Whitney test followed by Holm's correction for multiple testing. Adults-No Virus n=81, Adults-SARS-CoV-2 n=45, Adults-Other Virus n=28, Children-No Virus n=34, Children-SARS-CoV-2 n=38, and Children-Other Virus n=11.
