# Supplementary material for: Upper airway gene expression shows a more robust adaptive immune response to SARS-CoV-2 in children
Source: Nat Commun. 2022 Jul 8;13:3937. doi: 10.1038/s41467-022-31600-0 (PMC9263813; doi:10.1038/s41467-022-31600-0)
Supplement: Supplementary file 3 — Description of Additional Supplementary Files [file 41467_2022_31600_MOESM3_ESM.docx]

**Description of Additional Supplementary Files**

**Supplementary Data 1**. Sample metadata, including known clinical diagnoses.

**Supplementary Data 2**. Differential expression results between the SARS-CoV-2 and No Virus groups in adults and children separately.

**Supplementary Data 3**. GSEA applied to the differential expression results between the SARS-CoV-2 and No Virus groups in adults and children separately.

**Supplementary Data 4**. Differential expression results directly comparing adults and children with SARS-CoV-2, controlling for viral load.

**Supplementary Data 5**. GSEA applied to the direct comparison between adults and children with SARS-CoV-2.

**Supplementary Data 6.** *In silico* deconvolution of cell type proportions.
